# Inhibition of ABI2 ubiquitination-dependent degradation suppresses TNBC cell growth via down-regulating PI3K/Akt signaling pathway

**DOI:** 10.1186/s12935-024-03407-0

**Published:** 2024-06-27

**Authors:** Linlin Lv, Shujing Li, Jie Kang, Yulin Li, Nannan Zhao, Dongman Ye, Fengying Qin, Jing Sun, Tao Yu, Huijian Wu

**Affiliations:** 1 School of Bioengineering & Key Laboratory of Protein Modification and Disease, Dalian University of Technology, Dalian, Liaoning Province 116024 China; 2 Cancer Hospital of Dalian University of Technology, Shenyang, 110042 China; 3 Department of Pharmacy, First Affiliated Hospital of Dalian Medical University, Dalian, Liaoning China

**Keywords:** TNBC, ABI2, CBLC, PI3K/Akt, Colistimethate sodium

## Abstract

Triple negative breast cancer (TNBC) is a type of cancer that lacks receptor expression and has complex molecular mechanisms. Recent evidence shows that the ubiquitin-protease system is closely related to TNBC. In this study, we obtain a key ubiquitination regulatory substrate-ABI2 protein by bioinformatics methods, which is also closely related to the survival and prognosis of TNBC. Further, through a series of experiments, we demonstrated that ABI2 expressed at a low level in TNBC tumors, and it has the ability to control cell cycle and inhibit TNBC cell migration, invasion and proliferation. Molecular mechanism studies proved E3 ligase CBLC could increase the ubiquitination degradation of ABI2 protein. Meanwhile, RNA-seq and IP experiments indicated that ABI2, acting as a crucial factor of tumor suppression, can significantly inhibit PI3K/Akt signaling pathway via the interaction with Rho GTPase RAC1. Finally, based on TNBC drug target ABI2, we screened and found that FDA-approved drug Colistimethate sodium(CS) has significant potential in suppressing the proliferation of TNBC cells and inducing cell apoptosis, making it a promising candidate for impeding the progression of TNBC.

**Supplementary Information:**

The online version contains supplementary material available at 10.1186/s12935-024-03407-0.

## Introduction

Breast cancer is a condition where the growth of mammary epithelial cells becomes unregulated due to the influence of different cancer-causing agents. Breast cancer is currently the leading type of cancer among women in the United States, accounting for 31% of new cases [1], which has garnered significant global interest. By analyzing the data on Global Burden of Disease (GBD) in the previous phase, the research team discovered that the enduring impact of breast cancer had been growing. The worldwide age-standardized incidence rate (ASIR) has steadily risen from 21.44 per 100,000 individuals in 1990 to 24.17 per 100,000 individuals in 2019 [2]. Triple negative breast cancer (TNBC) is a prevalent form of breast cancer, constituting approximately 15–20% of newly-detected cases, characterized by the absence of the expression of estrogen receptor (ER), progesterone receptor (PR) and human epithelial growth factor receptor 2 (HER-2). Compared to other subtypes, TNBC is highly aggressive and metastatic with poorer prognosis and higher grading, which is more common among young women [3, 4]. The lack of receptor expression in TNBC leads to its insensitivity to targeted therapies, and its complex molecular mechanism causes serious chemotherapy resistance, all of which leads to the lack of effective clinical therapeutic drugs for this cancer. Therefore, it has become an urgent task to actively explore the molecular mechanism of TNBC and find better therapeutic drugs for patients in clinics.

The process of ubiquitin modification involves the covalent binding of one or more ubiquitin molecules to substrate protein molecules through a series of enzyme actions. Ubiquitination, a significant protein modification after translation, plays a crucial role in various intracellular processes including cell cycle progression, programmed cell deaths, DNA damage repair and the regulation of gene expression [5, 6]. With the deepening of research on ubiquitination modification, researchers have gradually turned to the ubiquitin-proteasome system, which has been as the target of tumor therapies, especially the development of novel anti-tumor drugs targeting ubiquitin ligase E3, proteasome and deubiquitinating enzymes, becoming a hot research topic at present. According to recent research, the enhancement of the effectiveness of cancer immunotherapy for TNBC can be achieved by targeting E3 ubiquitin ligase Cop1, which regulates the secretion of chemokines and the infiltration of macrophages in a tumor microenvironment [5]. EIF6-224aa is a new peptide that hinders the ubiquitin-proteasome pathway, leading to the stimulation of the Wnt/β-catenin pathway and the reduction of MYH9 degradation, thus it is a new promising prognostic and therapeutic target for TNBC treatment [7]. Hence, ubiquitination modification plays a crucial part in the genesis and progression of TNBC, potentially offering a hopeful treatment approach.

Despite numerous studies in which the correlation between ubiquitination and TNBC has been examined and dissected, there has been a lack of ubiquitination-related databases used by researchers to comprehensively screen and analyze the significant regulatory function of ubiquitination substrate proteins in TNBC. Therefore, in view of the importance of ubiquitination modification in TNBC, we used bioinformatic methods to screen 279 substrate proteins included in *hUbiquitome* (the first comprehensive database of human ubiquitination cascades) and obtained the protein ABL-interactor 2 (ABI2), which is closely related to the survival and prognosis of differentially-expressed triple-negative-subtype proteins.

ABI2 gene produces a protein that interacts with the ABL1 kinase, which is encoded by the ABL proto-oncogene-1. It can specifically bind to the carboxy-terminal proline-rich sequences of Src homology 3 (SH3) and ABL, which has been shown as one of the non-receptor tyrosine kinases involved in the regulation of intercellular signaling [8]. ABI2 protein has been demonstrated in previous research to have the ability to engage in intercellular signal transduction, ubiquitination modification, the regulation of cell cycles, epithelial-mesenchymal metastasis (EMT) and cell growth, either directly or indirectly, and it’s a promising new target of tumor therapies [9–12]. The study of Jensen et al., has shown ABI2, as a PIM1 substrate, can be phosphorylated at Ser183 by PIM1 and thereby enhanced wave regulatory complex (WRC) formation, resulting in increased protrusive activity and cancer cell motility [13]. Chen et al., proved that ABI2-mediated transcriptional axis MEOX2/KLF4-NANOG promotes liver cancer growth, metastasis and sorafenib resistance by maintaining the cancer stem cells population [12]. The paper of Huang et al., also indicated that ABI2, as a tumor suppressor and a cell migration inhibitor, drives epithelial-mesenchymal transition by activating c-JUN/SLUG signaling pathway [11]. However, the mechanisms of ABI2 involved in TNBC or its targeted intervention drugs have not been thoroughly studied. The aim of this study is to delve deeper into the functions of ABI2 in TNBC, which will help in identifying drug targets and potential drugs for treating TNBC in the future.

## Methods and materials

### Bioinformatic analysis

The *hUbiquitome* database (http://202.38.126.151/hmdd/hubi/) was used to gather all proteins serving as substrates for human ubiquitination. Subsequently, the expression of these genes in various molecular subtypes of breast cancers was examined utilizing The Cancer Genome Atlas (TCGA) database. Differentially expressed genes (DEGs) in TCGA were identified using TCGAbiolinks method, pair-wise tests and False Discovery Rate (FDR) correction, and the criteria for identifying these DEGs were set as FDR < 0.05 with |Log2FC| > 2. Furthermore, target genes with a low prognostic value (*p* < 0.05) were selected through a survival analysis of differentially-expressed genes from basal subtypes of TNBC. The *Ubibrowser* database (http://ubibrowser.bio-it.cn/ubibrowser/) was used to predict and screen the E3 ubiquitin ligases with high scores, which might interact with target proteins.

### Immunohistochemistry

A human tissue microarray involving 30 patients with paired TNBC (Cat No. HBreD180Bc01-1) was bought from Superbiotek Pharmaceutical Technology Co. Ltd. located in Shanghai, China. ABI2 immunohistochemistry (IHC) staining was conducted using established protocols, with a ABI2 antibody diluted at 1:100 (Santa Cruz, No. sc-393,982; Dallas, USA).

### Cell culture

Human normal breast cell line MCF-10 A, Luminal A breast cancer cell line MCF-7, human TNBC cell line MDA-MB-231 and human embryonic kidney cell line 293T (HEK293T) cells were purchased from American Type Culture Collection (ATCC; Manassas, USA) and cultured in DMEM containing 10% fetal bovine serum (FBS).

### Transfection experiments

To identify the gene functions of ABI2, MDA-MB-231 cells were seeded in 6-well plates for 48 h and then transfected for gene overexpression. Briefly, the transfected plasmid containing ABI2 were used for gene overexpression (OE-ABI2), and MDA-MB-231 cells transfected with empty vector were used as a negative control (NC). Pasmids were purchased from GenePharma (Shanghai, China).

### Cell viability and proliferation

To assess the growth and survival of those MDA-MB-231 cells, a total of 2,500 cells were placed in each well of 96-well plates, whose viability and proliferation were analyzed using the CCK-8 method and the light absorption was measured at the value of 450 nm.

### Cell migration

MDA-MB-231 cells were grown in 6-well plates until reaching a cell confluency of 95% ~ 100%, which were subsequently deprived of serum through incubation in a medium without serum for the entire night. Following the substitution of the new medium, a 200 µL pipet tip was utilized to generate a solitary scratch in the cellular monolayer of every well. Cells were imaged using a phase contrast microscope (Olympus Corp; Tokyo; Japan) at a magnification of 100 × within 0 and 24 h after the scratch. Before plating the cells, a mark was made on the bottom of those plates outside the well to establish a point of reference for the repeated imaging of cell migration. This method allows for the imaging of identical regions at all sampling time points.

### Cell invasion

The evaluation of cell invasion was conducted by employing a Transwell system containing polycarbonate membranes with 8 μm pores, which were coated with a matrigel matrix (manufactured by Corning Inc.; located in Corning, USA). In summary, the cells (5 × 10^4^) were diluted in 100 µL of media without serum and subsequently introduced into the upper chamber. The lower chamber was supplemented with 800 µL of DMEM containing 10% FBS. Following a 24-hour period, the infiltrated cells were immobilized using 10% methanol and subjected to staining with 0.1% crystal violet (Solarbio; Beijing, China). A microscope was used to capture the images, and the number of invading cells was tallied.

### TUNEL

MDA-MB-231 cells were grown in 6-well plates and treated with 0.1% Triton X-100 for a duration of 10 min. Afterwards, the incubated samples were washed with PBS, which were then treated with TUNEL-based apoptosis detection assay (Roche; Basel, Switzerland) kits following the manufacturer’s instructions. The average number of TUNEL-positive cells (green spots) was determined by counting apoptotic cells using a fluorescence microscope purchased from Olympus in Tokyo, Japan.

### Flow cytometry

The detection of cell apoptosis was performed utilizing a kit for apoptosis detection, specifically the Annexin V-FITC/PI obtained from Meilun in Dalian, China. Different culture media were added to 6-well plates to culture MDA-MB-231 cells for 48 h, which were initially treated with 0.25% trypsin and then rinsed twice with a PBS solution. Next, the gathered cells were treated with 5 µL of Annexin V-FITC and 5 µL of PI, which were then left to incubate at room temperature in the absence of light for a duration of 15 min. The stained cells were analyzed using flow cytometry (BD LSRFortessa™ Cell Analyzer; Franklin Lake, USA).

The measurement of the cell cycle was conducted by employing a cell cycle assay kit (Meilun; Dalian, China) in accordance with the guidelines provided by the manufacturer. MDA-MB-231 cells were collected, rinsed with PBS, and immobilized in ethanol for the duration of a whole night at 4 °C. Before analysis, the cells were rinsed once more with PBS, suspended and subjected to RNase A treatment with 10 µL for 30 min at 37 °C, and were subsequently incubated in the absence of light with 25 µL of PI for 30 min. Afterwards, the samples underwent an analysis using flow cytometry (BD LSRFortessa™ Cell Analyzer; Franklin Lake, USA).

### Co-IP

According to previous method [14], HEK293T cells were lysed using cell lysis buffer purchased from Solarbio in Beijing, China, and the supernatant was collected, which, next, was incubated at 4℃ overnight after the addition of ABI2 (1: 1,000; Santa Cruz, No. sc-393,982; Dallas, USA), CBLC (1: 1,000; Abcam, No. ab177956; Cambridge, UK), Flag (1: 2,000; Applygen, No. C1316; Beijing, China), HA (1: 2,000; Applygen, No. C1318; Beijing, China) or IgG (1: 2,000; Abcam, No. ab172730; Cambridge, UK) antibodies. Following this, the above liquid was subjected to incubation with protein A/G magnetic beads (MedChemExpress; Shanghai, China) at a temperature of 4℃ for a duration of 4 h. Then, the magnetic beads were isolated, and the above liquid was gathered. Subsequently, the Pierce IP lysis buffer (Thermo; MA, USA) was introduced, followed by sample heating at 100 °C for 7 min. The samples were then subjected to a western blotting analysis.

### Ubiquitylation assay

GFP-CBLC, HA-CBLC, Flag-ABI2 and HA-Ub plasmids were introduced into MDA-MB-231 or HEK293T cells, followed by immunoprecipitation using anti-HA, anti-Flag affinity gel to isolate the ubiquitinated ABI2 from specified cells. Moreover, proteasome inhibitor MG132 was used to detect the ubiquitination of ABI2 by CBLC. The co-incubated samples were finally analyzed using western blotting. For detailed methods, please refer to previous articles published by our research group [15, 16].

### Immunoprecipitation (IP) and LC-MS/MS analysis

According to previous method [17], MDA-MB-231 cells overexpressing GFP-ABI2 were harvested and lysed. Anti-GFP or IgG antibodies were added to the lysis solution for antibody immobilization. After incubation with Protein A/G Magnetic Beads, the protein complex was centrifuged and then washed with Pierce IP Lysis Buffer for SDS-PAGE analysis. In addition, LC-MS/MS analysis was implemented to detect the polypeptide sequence of protein samples. Finally, the polypeptide sequence was identified using ProteinPilot software of the AB SCIEX Triple TOF™ 5600 plus MS system (MA, USA).

### Quantitative real-time PCR analysis

RNAex Pro RNA reagent purchased from Accurate Biology in Hunan, China was utilized for the isolation and extraction of total cell RNA. Subsequently, the synthesis of cDNA was performed using an Evo M-MLV RT MIX kit, also purchased from Accurate Biology in Hunan, China. The mRNA expression level of various genes was quantified using a SYBR^®^ green premix Pro Taq HS qPCR kit (Accurate Biology; Hunan, China) in an ABI 7500 real-time PCR system (Applied Biosystems; CA, USA). Table 1 displays the primer sequences.

**Table 1:** Primer sequences used for qPCR assay

Gene	Primer sequence (5’ to 3’)
*fgf12*	F: TCTATTCTTCCACACTGTACCG; R: GCTTGGTTTTCTTCACTCTGTT
*il-6*	F: CACTGGTCTTTTGGAGTTTGAG; R: GGACTTTTGTACTCATCTGCAC
*csf1*	F: TGATTGACAGTCAGATGGAGAC; R: TAGCACACTGGATCTTTCAACT
*itgb4*	F: AATGGGGGCATCTGTAATGG; R: GAGTAGTTGATCTCGCAGATGG
*il-4r*	F: TACTTGCGAGTGGAAGATGAAT; R: TATAGTTATCCGCACTGACCAC
*ppp2r2a*	F: AGACATAACCCTAGAAGCATCG; R: GTTTGTAGTAGCTACGGCAATG
*apc2*	F: CTGAAGCACCTACAGGGAAAA; R: CTGGAACTTGAGGTTGTACAGG
*ppp2r1a*	F: AACTTCGACAGTACTTCCGGAA; R: ATGATCTCACTCTTGACGTTGT
*wnt10b*	F: TTCAGGGTCTGCACATCG; R: GGAAAAAGCACTTTCTCGGAAA
*id1*	F: CTACGACATGAACGGCTGTTA; R: CAACTGAAGGTCCCTGATGTAG
*atf2*	F: ATTAAAAGCTGCTTTGACCCAG; R: TCATCAGGATCTTCGTTAGCTG
*pdgfd*	F: CAATGATGATGCCAAGCGTTACAG; R: AGTTCCACAGCCACAATTTCCTC
*fgf9*	F: ACCCAAGAGTGTGTATTCAGAG; R: AGTGTCCACGTGCTTATATAGG
*hsp90aa1*	F: CCAGTTCGGTGTTGGTTTTTAT; R: CAGTTTGGTCTTCTTTCAGGTG
*angpt1*	F: GGGAGGTTGGACTGTAATACAA; R: TGTCATACTGTGAATAGGCTCG
*itgav*	F: ACAGGCAATAGAGATTATGCCA; R: TTTATCCTGTTTCGACCTCACA
*ccnd2*	F: TTTAAGTTTGCCATGTACCCAC; R: ACGTCTGTGTTGGTGATCTTAG
*bmp6*	F: CATGGTCATGAGCTTTGTGAAC; R: TGAACTTGAACTCTTTGTGGTG
*wtip*	F: GGCTGCGAGACAACCATCC; R: GGCAACGACGACACAGTAGG
*spp1*	F: AATGCTGTGTCCTCTGAAGAAACC; R: AGTCAATGGAGTCCTGGCTGTC
*itga10*	F: TTACAATACGAGCCTGAGTCTC; R: GCAGCTAAACTCAAACTCTAGC
*csf3*	F: TTAGAGCAAGTGAGGAAGATCC; R: CCATTCCCAGTTCTTCCATCT
*csf1r*	F: CATATGGACATTCATCAACGGC; R: GCCTCATCACACCTATCAGTG
*gapdh*	F: TGAAGGTCGGAGTCAACGG; R: CCTGGAAGATGGTGATGGG

### Western blotting

Protein content was obtained from MDA-MB-231 cells, which were then isolated and divided using SDS-PAGE, and subsequently transferred onto PVDF membranes (Millipore; MA, USA). After being sealed with a blocking buffer containing 5% BSA, the membranes were incubated overnight at 4℃ with primary antibodies ABI2 (1: 1,000; Santa Cruz, No. sc-393,982; Dallas, USA), CBLC (1: 1,000; Abcam, No. ab177956; Cambridge, UK), PI3K (1: 1,000; CST, No. 4292; Danvers, USA), p-PI3K (1: 1,000; CST, No. 17,366; Danvers, USA), Akt (1: 1,000; CST, No. 4691; Danvers, USA), p-Akt (1: 1,000; CST, No. 4060; Danvers, USA), mTOR (1: 1,000; CST, No. 2983; Danvers, USA), p-mTOR (1: 1,000; CST, No. 5536; Danvers, USA) and GAPDH (1: 5,000; CST, No. 5174; Danvers, USA), which were subsequently incubated at room temperature for 1 h with a secondary antibody (1: 5,000; Abbkine, A21020; Wuhan, China). In the end, the protein bands were observed using an imaging device (Tanon 4200; Shanghai, China).

### Transcriptomic analysis

By employing the polyA structure located at the 3ʹ-terminal end of messenger RNA and associated molecular biology techniques, we isolated the entire RNA of MDA-MB-231 cells, which was then subjected to mRNA isolation, fragmentation, the synthesis of double-stranded cDNA, the modification of cDNA fragmentation, purification using magnetic beads, the sorting of fragments and the expansion of library. Following the completion of quality control, a sequencing library compatible with the Illumina platform was ultimately acquired. RNA-seq data was analyzed for gene structure, expression level, expression differences and gene enrichment, etc. The DESeq method was employed to perform a comparison analysis between the two groups, and the criteria for identifying differentially-expressed genes were set as q value < 0.05 and fold change ≥ 2 or fold change ≤ 0.5. A bioinformatics analysis including the KEGG pathway of the differentially-expressed genes was conducted.

### Molecular docking and virtual screening

Molecular docking simulation and prediction of the binding affinity of CBLC for ABI2 protein were performed using ClusPro [18] for protein-protein docking. The top 10 cluster centers with the largest number of cluster members were then retrieved and inspected visually one by one. A further evaluation was conducted on the intermolecular interactions at the most probable positions. MOE v2018.01 was used to analyze the docked formations and interface residues. In addition, virtual screening was conducted using MOE. A total of 2800 drugs from the FDA drug library, which were approved drug molecules, were chosen as the virtual screening library. The structure of ABI2 was defined as a receptor. The site of the ABI2 protein binding to the CBLC protein was defined as the small-molecule binding pocket site. A monomer model of ABI2 was used as the receptor, and the drug compound library was used as the virtual screening library. Following the adaptable docking process, the molecules receiving scores were chosen and categorized into structural clusters through fingerprint-based clustering. Eventually, the top 9 molecules were recognized as potential hits.

### Statistical analysis

The data was presented as the mean ± SD. GraphPad Prism 6.0 software was utilized for all analyses. One-way ANOVA was used to determine differences among multiple groups, while an unpaired student’s t-test was used to analyze differences between each two groups. Statistically-significant values were those less than 0.05 or 0.01.

## Results

### The expression of ABI2 is markedly reduced in patients with TNBC

**Fig. 1:**
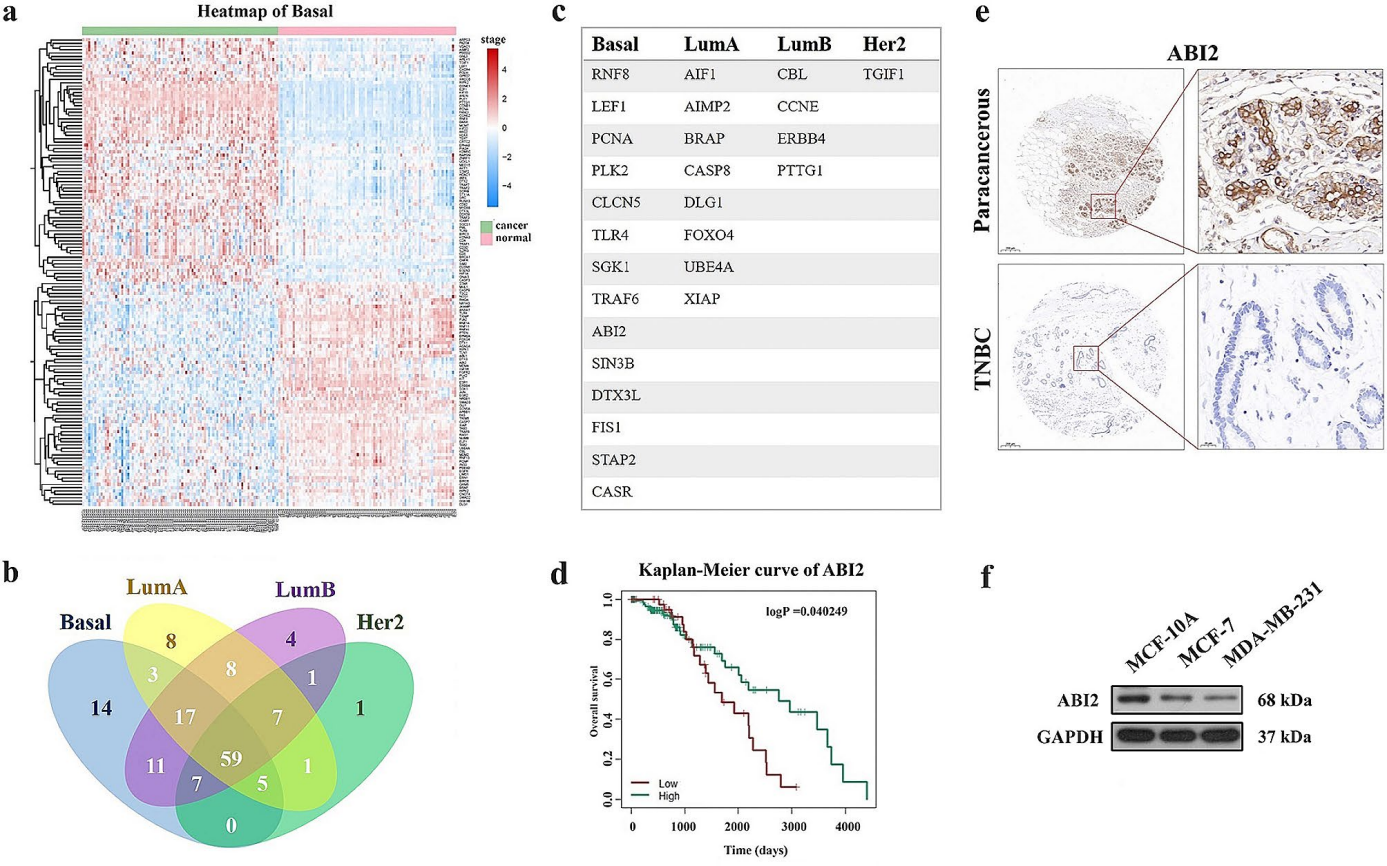
The expression of ABI2 is markedly reduced in patients with TNBC. (**a**) Heatmap analysis of the 279 ubiquitinated human substrate proteins expression in Basal subtype of breast cancer; (**b**) Venn diagram analysis of differentially-expressed substrate proteins in Basal, LumA, LumB and Her2 subtypes of breast cancer; (**c**) The 14 distinctively-expressed proteins in the Basal subtype of breast cancer; (**d**) Kaplan- Meier curve of ABI2 in the survival analysis of patients with TNBC. (**e**) IHC analysis of ABI2 protein expression in TNBC tumors. (**f**) Western blotting analysis of ABI2 protein expression in MDA-MB-231, MCF-10 A and MCF-7 cells

In view of the importance of ubiquitination modification in TNBC, we obtained 279 ubiquitinated human substrate proteins from *Ubiquitome* database; and they were then analyzed based on the TCGA database to determine their expression in various molecular subtypes of breast cancers, including Basal, LumA, LumB and Her2 (Fig. 1a and Fig. S1). Moreover, the differentially-expressed proteins were screened out, with a Venn diagram drawn (Fig. 1b). Briefly, Fig. 1c displays the findings that identify a collective of 14 distinctively-expressed proteins, namely RNF8, LEF1, PCNA, PLK2, CLCN5, TLR4, SGK1, TRAF6, ABI2, SIN3B, DTX3L, FIS1, STAP2 and CASR, which were exclusively presented in the Basal subtype of breast cancer (TNBC). In addition, an analysis of the survival of these proteins in TNBC was conducted, with the criteria for significance being *p* < 0.05. As shown in Fig. 1d, result indicated that ABI2 was the key regulatory factor in the survival curve of patients with TNBC. Moreover, ABI2 expression was positively correlated with the survival rate, but its functions in TNBC were still unclear.

In order to investigate the expression level of ABI2 protein, an IHC analysis was conducted on a tissue microarray consisting of 30 pairs of TNBC tumor tissues and their corresponding adjacent tissues. The results revealed a significant decrease of ABI2 protein expression in TNBC tumors compared to adjacent non-cancerous tissues. Additionally, ABI2 was predominantly localized in the cytoplasm, as depicted in Fig. 1e. Furthermore, in vitro experiments, human normal breast cell line (MCF-10 A cells), Luminal A breast cancer cell line (MCF-7 cells) and TNBC cell line (MDA-MB-231 cells) were selected for ABI2 protein expression verification, and the results indicated that ABI2 protein expression in MDA-MB-231 cells was notably lower than that in MCF-10 A cells and MCF-7 cells (Fig. 1f). Hence, the expression of ABI2 was markedly reduced in individuals with TNBC and exhibited a positive association with the rate of survival.

### AB12 inhibits cell viability, migration, invasion and arrests cell cycle of TNBC cells

**Fig. 2:**
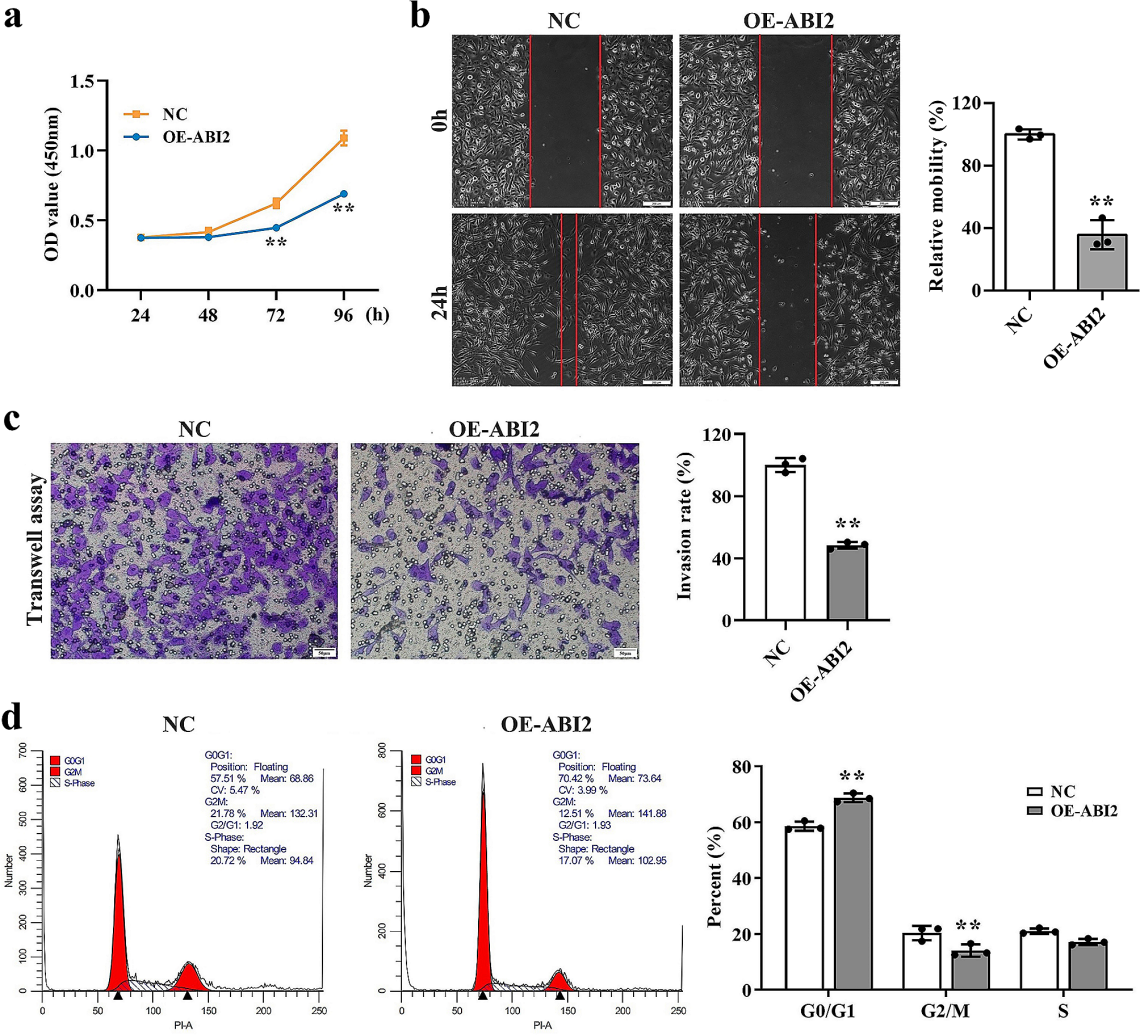
AB12 inhibits cell viability, migration, invasion and arrests cell cycle of TNBC cells. (**a**) Effects of OE-ABI2 on the growth pattern of MDA-MB-231 cells detected by CCK-8 method; (**b**) Effects of OE-ABI2 on the cell migration of MDA-MB-231 cells detected by wound-healing assay; (**c**) Effects of OE-ABI2 on the cell invasion of MDA-MB-231 cells detected by Transwell assay; (**d**) Effects of OE-ABI2 on the cell cycle of MDA-MB-231 cells detected by flow cytometry. Data are presented as the mean ± SD. **p < 0.01 vs. NC group

To investigate the effects of ABI2 protein on TNBC cells, we conducted a range of in vitro phenotypic experiments. As shown in Fig. 2a, CCK8 method was employed to observe the effects of OE-ABI2 on the growth pattern of MDA-MB-231 cells. The findings revealed that the overexpression of ABI2 (OE-ABI2 group) suppressed the proliferation of TNBC cells in comparison to the NC group over a period of 72 to 96 h. Meanwhile, the cell viability is no obvious difference between OE-ABI2 and NC groups when the transfection time less than 48 h. Moreover, the effects of ABI2 on cell migration and invasion in MDA-MB-231 cells were examined using wound-healing and Transwell assays after 24 h transfection. According to Fig. 2b, the migration of MDA-MB-231 cells in OE-ABI2 group was effectively suppressed compared to NC group. Moreover, compared with NC group, the invasion of MDA-MB-231 cells was significantly inhibited by in OE-ABI2 according to the result in Fig. 2c. Given the important role of ABI2 in cell cycles, we detected the cell cycle via flow cytometry. As shown in Fig. 2d, OE-ABI2 group showed a significant increase in the percentage of cells in the G0/G1 phase and a notable decrease in the G2/M phase, while no significant changes were observed in the S phase compared to NC cells, suggesting that ABI2 arrested MDA-MB-231 cells at the G0/G1 phase. Hence, these findings suggested that ABI2 hindered the proliferation of TNBC cells by halting the cell cycle at the G0/G1 stage. Therefore, ABI2 has notably inhibitory effects on TNBC cells through decreasing or halting the cell viability, cell migration and invasion, and cell cycle.

### ABI2 inhibits PI3K/Akt signaling pathway in TNBC cells

**Fig. 3:**
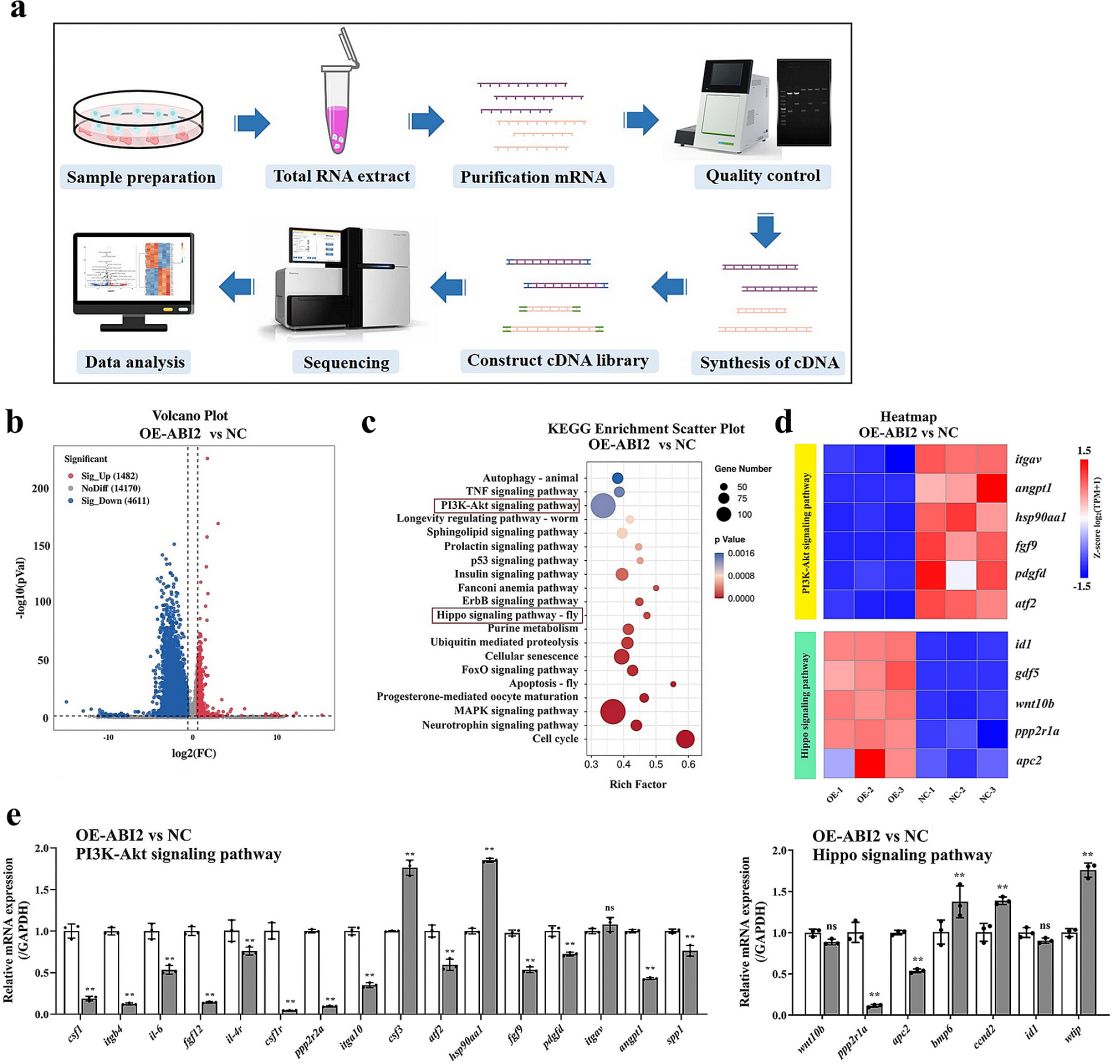
ABI2 inhibits PI3K/Akt signaling pathway in TNBC cells. (**a**) Flowchart of RNA-seq analysis on MDA-MB-231 cells treated with OE-ABI2; (**b**) Volcano diagram analysis of differentially expressed genes in MDA-MB-231 cells with OE-ABI2; (**c**) KEGG analysis of differentially expressed genes in MDA-MB-231 cells with OE-ABI2; (**d**) Heatmap analysis of differentially expressed genes of PI3K/Akt and Hippo signaling pathways in MDA-MB-231 cells with OE-ABI2; (**e**) qPCR validation of differentially expressed genes of PI3K/Akt and Hippo signaling pathways in MDA-MB-231 cells with OE-ABI2. Data are presented as the mean ± SD. *p < 0.05, **p < 0.01 vs. NC group

In order to investigate the particular molecular processes controlled by ABI2 in TNBC cells, we performed RNA-seq analysis on MDA-MB-231 cells treated with OE-ABI2 in comparison to NC cells (Fig. 3a). According to the data presented in Fig. 3b, a total of 6093 genes were found to be different between MDA-MB-231 cells treated with OE-ABI2 and NC TNBC cells. Among these genes, 1482 showed an increase in expression while 4611 showed a decrease. In this RNA-seq study, the finding suggested that the PI3K/Akt and Hippo signaling pathways were enriched by ABI2, as revealed by KEGG analysis (Fig. 3c). The heatmap in Fig. 3d displays significant differential genes associated with the PI3K/Akt and Hippo signaling pathways, which were confirmed through qPCR in MDA-MB-231 cells. As shown in Fig. 3e, qPCR results showed that the expressions of PI3K/Akt signaling pathway-related genes including *csf1, itgb4, il-6, fgf12, il-4r, csf1r, ppp2r2a, itga10, atf2, fgf9, pdgfd, angpt1 and spp1* genes were in accordance with the RNA-seq result, which indicated that ABI2 possess inhibitory actions on PI3K/Akt signaling pathway. Hence, ABI2 inhibits PI3K/Akt signaling pathway in TNBC cells.

### ABI2 may regulate PI3K/Akt signaling pathway through interacting with RAC1 protein in TNBC cells

**Fig. 4:**
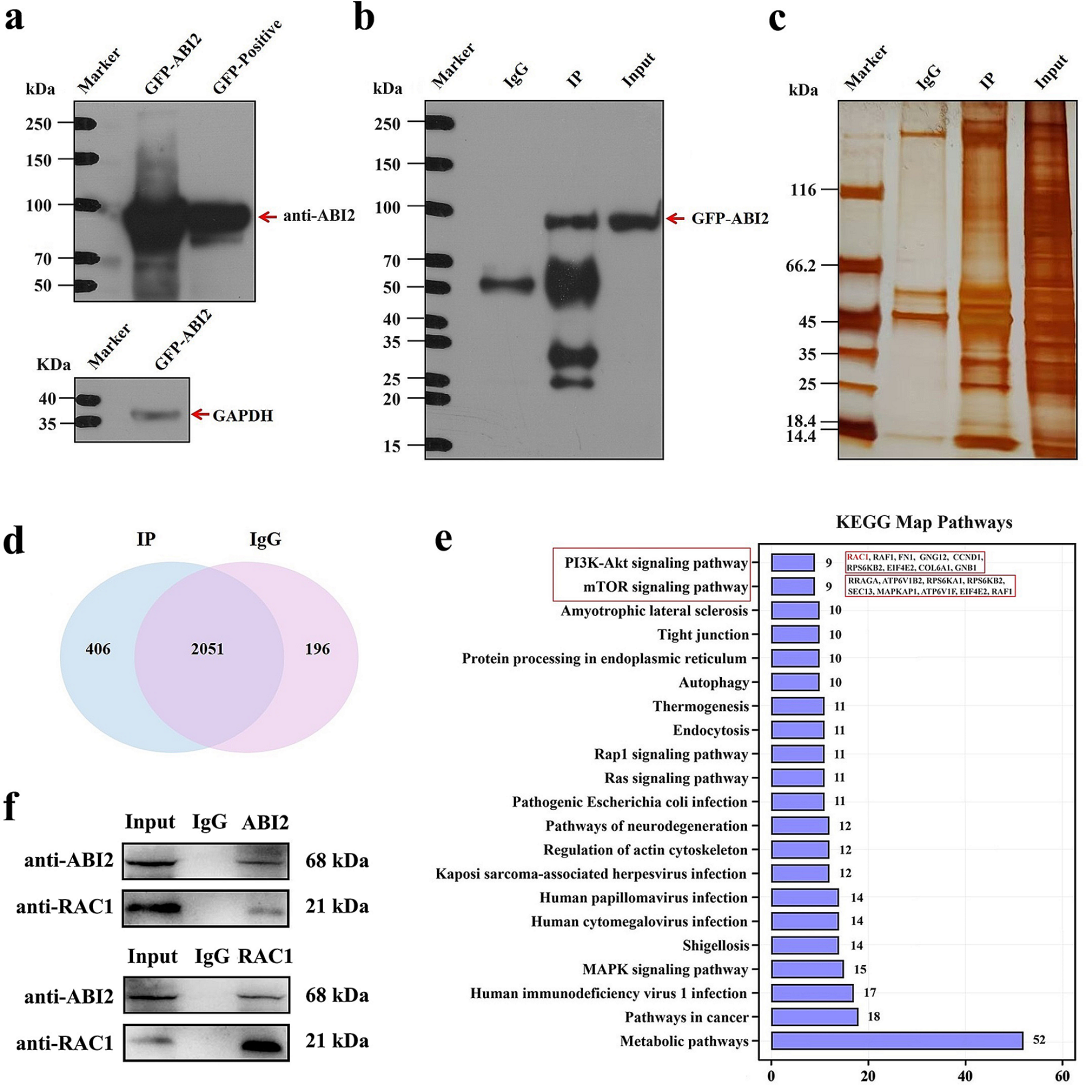
ABI2 may regulate PI3K/Akt signaling pathway through interacting with RAC1 protein in TNBC cells. (**a**) ABI2 protein expression validation in MDA-MB-231 cells; (**b**) ABI2 protein expression after IP experiment; (**c**) The silver staining of proteins in IP experimental group and IgG control group; (**d**) Venn diagram of differential proteins between IP experimental group and IgG control group; (**e**) KEGG analysis of signaling pathways involved in the differential proteins; (**f**) Co-IP results of the interaction between ABI2 and RAC1 proteins

To investigate the target proteins of ABI2 regulating the PI3K/Akt signaling pathway, we conducted an IP experiment with ABI2. As shown in Fig. 4a, we firstly detected ABI2 expression in OE-ABI2 treated MDA-MB-231 cells through western blotting, which confirmed the successful overexpression of ABI2 in MDA-MB-231 cells. Furthermore, as shown in Fig. 4b, ABI2 protein was detected through western blotting after IP experiment. Meanwhile, the silver staining results showed that the difference in protein levels between the IP experimental group and the IgG control group was significant (Fig. 4c). We further used LC-MS/MS to analyze the binding proteins of ABI2, and the results showed that a total of 406 ABI2-specific binding proteins were identified (Fig. 4d). In order to explore the signaling pathway involved in these differential proteins, we performed KEGG analysis, and the results indicated that ABI2 has an interaction with PI3K/Akt signaling (mainly includes COL6A1, FN1, GNB1, GNG12, RAC1, RAF1, CCND1, RPS6KB2 and EIF4E2) and mTOR signaling (mainly includes RRAGA, ATP6V1B2, RAF1, RPS6KA1, RPS6KB2, Sect. 13, MAPKAP1, ATP6V1F, EIF4E2) related proteins in TNBC cells (Fig. 4e). mTOR pathway is a downstream signal of PI3K/Akt, and they are crucial intracellular route that enhances cell proliferation, growth, tumorigenesis and motility. Among them, Rho GTPase RAC1 can bind with PI3K p110α isoform and thereby activate PI3K phosphorylation. As shown in Fig. 4f, Co-IP result confirmed the interaction between ABI2 and RAC1 proteins. Therefore, ABI2 may regulate PI3K/Akt signaling pathway through interacting with RAC1 protein in TNBC cells.

### Up-regulation of ABI2 via inhibiting CBLC mediated-ubiquitination may be potential treatment for TNBC

**Fig. 5:**
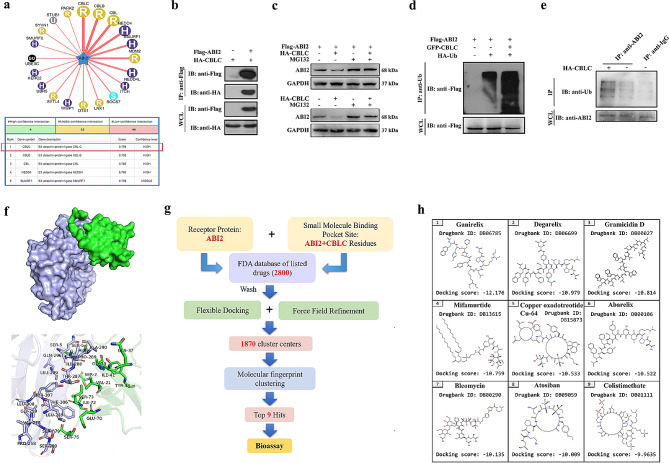
Up-regulation of ABI2 via inhibiting CBLC mediated-ubiquitination may be potential treatment for TNBC. (**a**) CBLC was predicted to be the E3 ligases of ABI2 protein by using bioinformatics; (**b**) There was a significant co-precipitation between the natural forms of ABI2 and CBLC in HEK293T cells through the employment of the Co-IP technique; (**c**) The exogenous and endogenous ubiquitination experiments with or without MG132. (**d-e**) CBLC could promote the binding of ABI2 to Ub and increase the ubiquitization degradation of ABI2 protein at both exogenous and endogenous levels of ubiquitination; (**f**) Molecular docking mode and amino acid interaction residues of ABI2 and CBLC proteins; (**g**) Virtual screening process from 2,800 FDA-approved drug molecules based on the molecular docking mode of ABI2 and CBLC proteins; (**h**) Top 9 molecules with the best ranking between ABI2 and CBLC proteins

In order to elucidate the ABI2 protein in TNBC, the *Ubibrowser* database was utilized to forecast the E3 ligases interacting with ABI2. As shown in Fig. 5a, the findings indicated that CBLC exhibited the greatest assurance, scoring 0.798. Relying on the noted correlation between ABI2 and CBLC in the field of bioinformatics, we successfully identified a significant co-precipitation between the natural forms of ABI2 and CBLC in HEK293T cells through the employment of the Co-IP technique (Fig. 5b). Furthermore, this discovery further validated the role of CBLC E3 ligase in facilitating the ubiquitization modification of ABI2 protein. The exogenous and endogenous ubiquitination experiments with or without MG132 in Fig. 5c had shown that proteasome inhibitor MG132 could inhibit ABI2 ubiquitination mediated by CBLC. Moreover, as shown in Fig. 5d-e, the results suggested that CBLC could promote the binding of ABI2 to ubiquitin (Ub) and increase the ubiquitization degradation of ABI2 protein at both exogenous and endogenous levels of ubiquitination. Furthermore, a simulation of docking was performed to investigate the amalgamation of ABI2 and CBLC proteins. The interaction between ABI2 and CBLC proteins is shown in Fig. 5f and Fig.S2a, where the Zdock’s docking score is 1,277.305. Studies on docking simulation have indicated that a large number of hydrophobic interactions and hydrogen bonds can be formed by residues around the interaction interface, which help stabilize protein-protein complexes. Tyr307, Phe306, Leu285, Val278, Ser279, Ser280, Peo258, Leu308, Gly259, Ile288, Thr287, Ala290, Pro289, Gln296 and Leu299 in CBLC protein interact with Ser73, Ile72, Ser76, Glu70, Gln37, Ser2, Gly39, Val21, Ile41, Tyr43, Ser6 and Ser5 in ABI2 protein, forming their hydrophobic surfaces. Hydrogen bonds were formed between Arg269, Lys292, Glu300, Asp304 and Lys303 of CBLC protein and Glu38, Gly7, Lys19, Gly74, Gly78, Ser77 and Ser3 of ABI2 protein respectively, whose length was 2.2Å, 2.2Å, 3.2Å, 2.6Å, 3.4Å, 3.5Å and 2.8Å respectively. The above residues may be active residues for ABI2-CBLC proteins interaction. In addition, clinical trials of drugs have been completed by the Food and Drug Administration (FDA) in general pharmacology, pharmacokinetics and toxicology, which lead FDA-based drug repositioning to be able to accelerate the drug research process. Therefore, as shown in Fig. 5g, we selected 2,800 FDA-approved drug molecules for our screening library; and ABI2 protein was characterized as a receptor, with its binding site on CBLC protein identified as a site for the binding of small molecules. Then, through matching, optimization, scoring and screening, as shown in Fig. 5h, the top 9 molecules with the best ranking were finally identified as potential hit molecules, which may be potential therapeutic drug for TNBC via the up-regulation of ABI2 protein expression through inhibiting CBLC E3 Ligase mediated-ubiquitination degradation.

### Colistimethate sodium (CS) suppresses TNBC cell growth through inhibiting ABI2-CBLC protein interaction

**Fig. 6:**
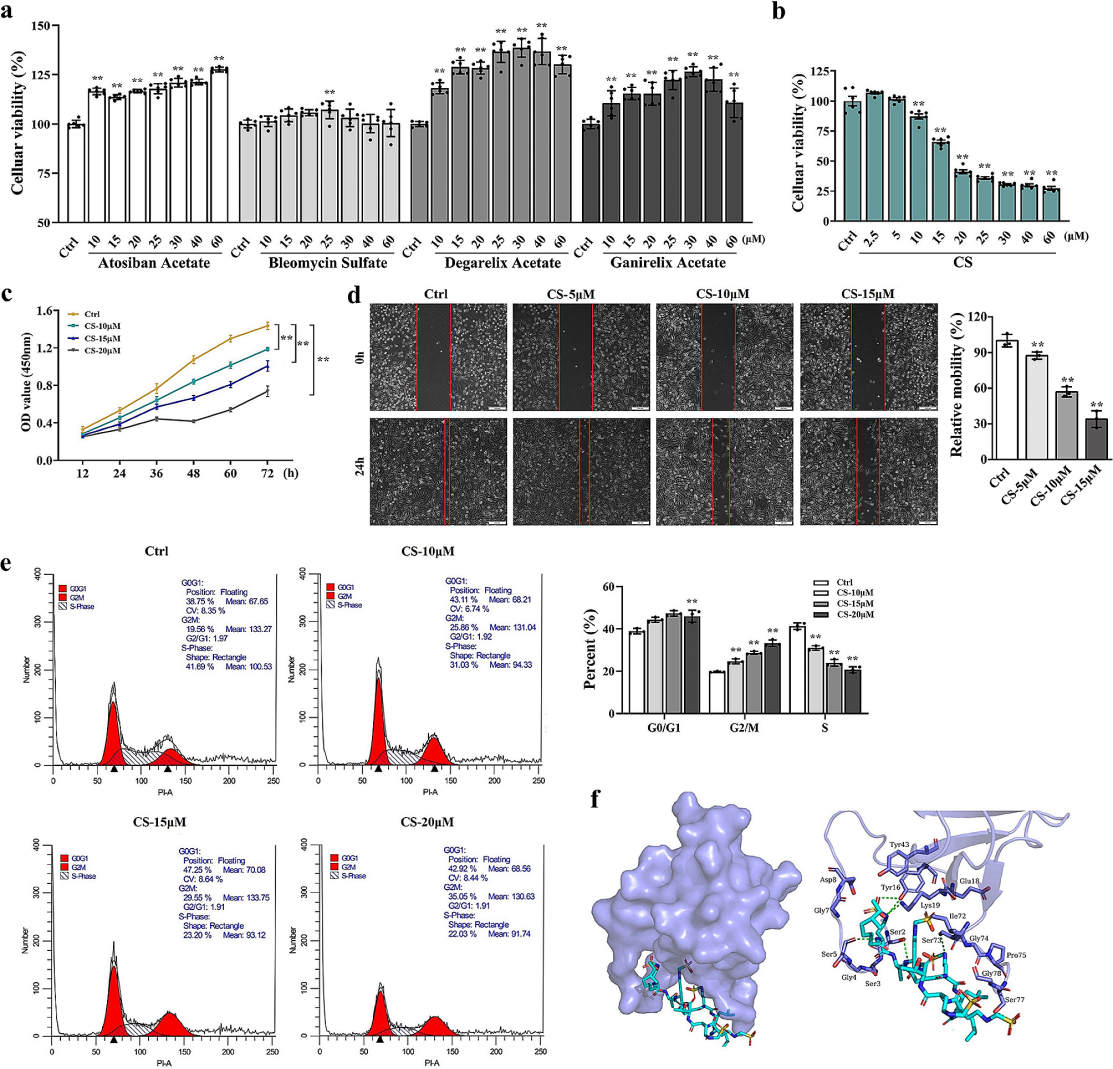
CS suppresses TNBC cell growth through inhibiting ABI2-CBLC protein interaction. (**a-b**) Effects of top molecules including Atosiban Acetate, Bleomycin Sulfate, Degarelix Acetate, Ganirelix Acetate and CS on MDA-MB-231 cells detected by CCK-8 method; (**c**) Effects of CS on the growth pattern of MDA-MB-231 cells detected by CCK-8 method; (**d**) Effects of CS on the cell migration of MDA-MB-231 cells detected by wound-healing assay; (**e**) Effects of CS on the cell cycle of MDA-MB-231 cells detected by flow cytometry; (**f**) Molecular docking mode and amino acid interaction residues of ABI2 protein and CS. Data are presented as the mean ± SD. *p < 0.05, **p < 0.01 vs. Ctrl group

In order to evaluate the inhibitory effect of small-molecule inhibitors targeting ABI2-CBLC protein interactions, the top molecules (Atosiban Acetate, Bleomycin Sulfate, Degarelix Acetate, Ganirelix Acetate and CS) were selected with cell viability determined using CCK8 assay (Fig. 6a-b). The findings indicated that CS suppressed the proliferation of MDA-MB-231 cells in a dose-dependent fashion. Moreover, according to an analysis of cell viability, CS could significantly inhibit cell viability when CS ≥ 10µM, while it had no notable effect on cell viability when CS ≤ 5µM. To examine the impact of CS on the growth of MDA-MB-231 cells at various time intervals (12, 24, 36, 48, 60 and 72 h), CS concentrations of 10, 15 and 20µM were chosen. As shown in Fig. 6c, the results showed that the OD450nm value gradually increased with the extension of time, but the higher the concentration of CS was, the lower the OD450nm value at each time point would be compared to the control (Ctrl) group. In addition, compared to Ctrl group, the migration of MDA-MB-231 cells were effectively suppressed by CS at the concentrations of 5, 10 and 15µM (Fig. 6d).

Flow cytometry was employed to identify the cell cycle, revealing that MDA-MB-231 cells exhibited negligible alterations in the G0/G1 phase following CS pre-treatment. However, a substantial rise in the cell percentage at the G2/M phase and a noticeable decline in the S phase were observed when the CS concentration reached or exceeded 10µM (Fig. 6e). These findings strongly suggested that CS had the capability to impede the progression of MDA-MB-231 cells in the G2/M phase. Therefore, CS may be a potent therapeutic drug against TNBC by inhibiting cell growth and proliferation via the arrest of cell cycles. The interaction between ABI2 protein and CS is shown in Fig. 6f and Fig.S2b. The hydrogen bond and hydrophobic interaction were formed between ABI2 and CS, and the binding sites of the two were formed and suitable stereocomplementary. The four oxygen atoms of CS form hydrogen bonds with Ser77, Ser2, Tyr16 and Lys19 in ABI2 protein. Its three nitrogen atoms form hydrogen bonds with Ser73, Ser5 and Tyr16 in ABI2 protein. CS interacts hydrophobic with residues Gly78, Ile72, Pro75, Gly4, Gly74, Ser3, Glu18, Gly7, Tyr43 and Asp8 in ABI2 protein. These interactions mainly contribute to the binding energy between ABI2 protein and CS. Therefore, CS might suppress TNBC cell growth through inhibiting ABI2-CBLC protein interaction.

### CS inhibits PI3K/Akt signaling pathway in TNBC cells

**Fig. 7:**
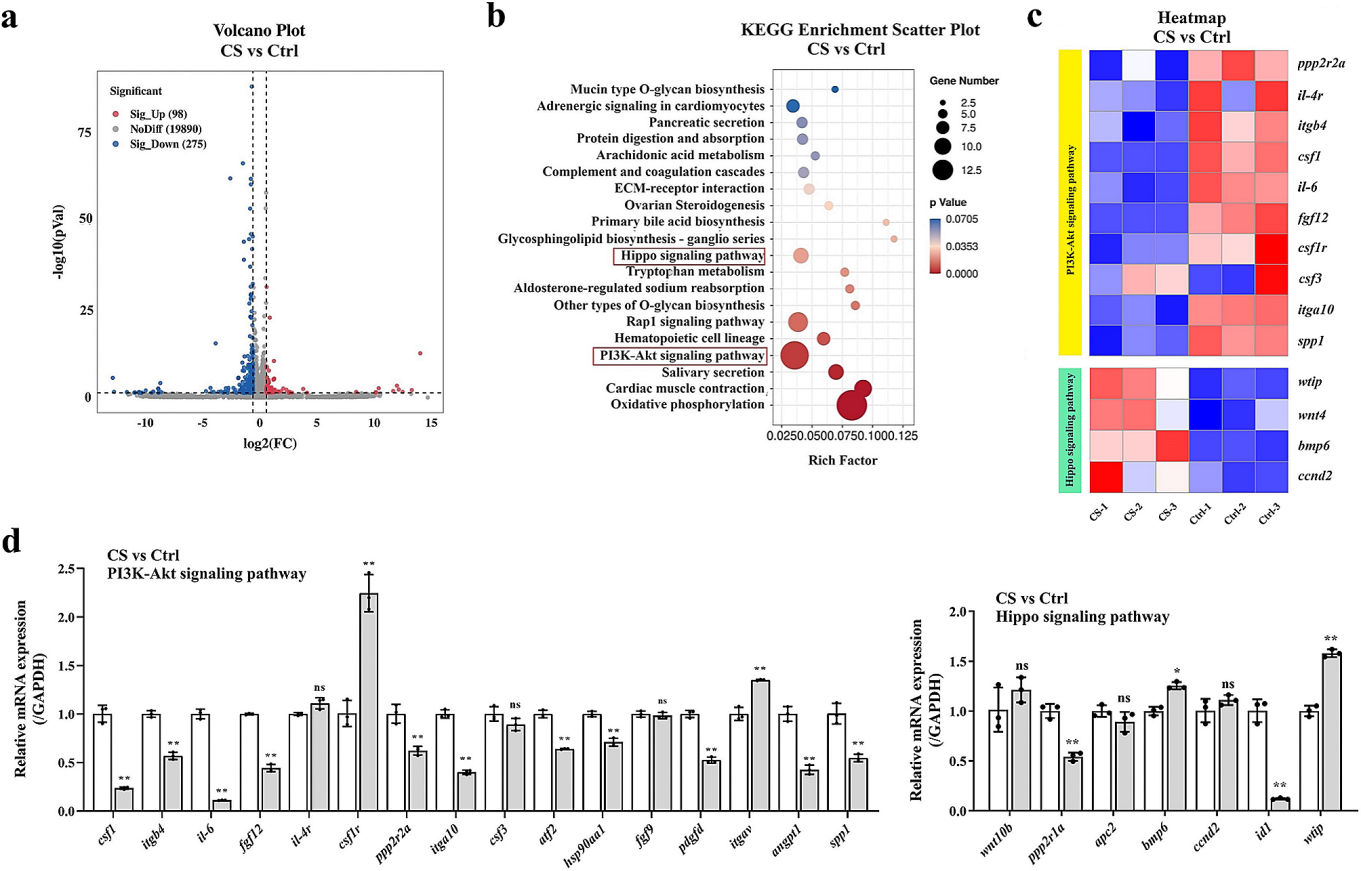
CS inhibits PI3K/Akt signaling pathway in TNBC cells. (**a**) Volcano diagram analysis of differentially expressed genes in MDA-MB-231 cells with CS; (**b**) KEGG analysis of differentially expressed genes in MDA-MB-231 cells with CS; (**c**) Heatmap analysis of differentially expressed genes of PI3K/Akt and Hippo signaling pathways in MDA-MB-231 cells with CS; (**d**) qPCR validation of differentially expressed genes of PI3K/Akt and Hippo signaling pathways in MDA-MB-231 cells with CS. Data are presented as the mean ± SD. *p < 0.05, **p < 0.01 vs. NC group

To investigate the molecular mechanism of CS inhibiting the growth of TNBC cells, we performed RNA-seq analysis on MDA-MB-231 cells treated with CS at the dose of 20 µM. According to the data presented in Fig. 7a, the findings indicated the presence of 373 genes with a differential expression in MDA-MB-231 cells with CS compared to Ctrl TNBC cells, consisting of 98 up-regulated genes and 275 down-regulated ones. In this RNA-seq study, the finding suggested that the PI3K/Akt and Hippo signaling pathways were enriched by CS, as revealed by KEGG analysis (Fig. 7b). The heatmap in Fig. 7c displays significant differential genes associated with the PI3K/Akt and Hippo signaling pathways, which were confirmed through qPCR in MDA-MB-231 cells. As shown in Fig. 7d, qPCR results also indicated that CS inhibited PI3K/Akt signaling pathway in TNBC cells.

### CS induces TNBC cells apoptosis via inhibition of PI3K/Akt signaling pathway mediated by ABI2

**Fig. 8:**
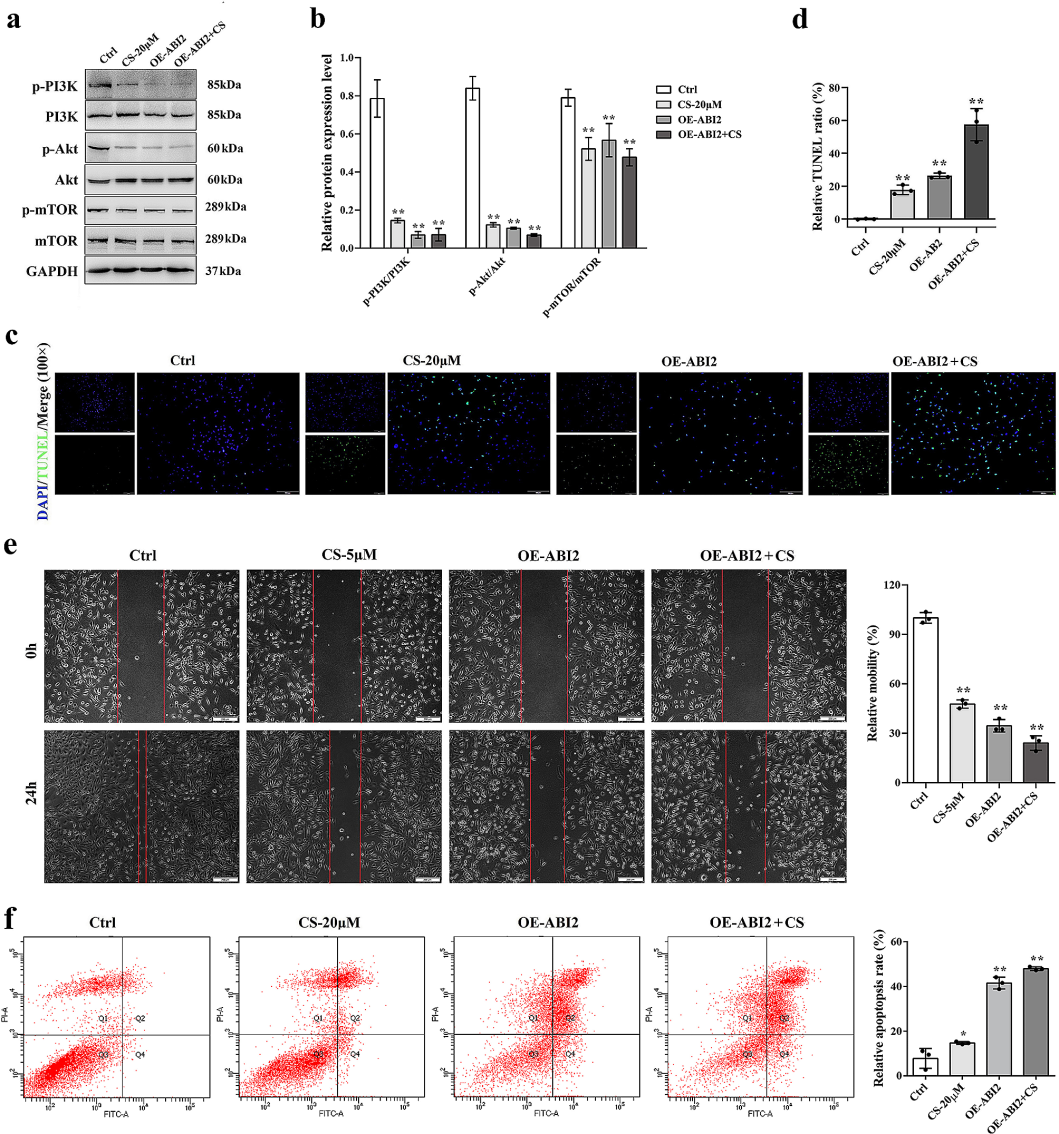
CS induces TNBC cells apoptosis via inhibition of PI3K/Akt signaling pathway. (**a-b**) Effects of CS and OE-ABI2 on PI3K/Akt signaling pathway of MDA-MB-231 cells detected by western blotting assay; (**c-d**) Effects of CS and OE-ABI2 on the apoptosis of MDA-MB-231 cells detected by TUNEL method; (**e**) Effects of CS and OE-ABI2 on the cell migration of MDA-MB-231 cells detected by wound-healing assay; (**f**) Effects of CS and OE-ABI2 on the apoptosis of MDA-MB-231 cells detected by flow cytometry. Data are presented as the mean ± SD. *p < 0.05, **p < 0.01 vs. Ctrl group

In order to further verify whether CS works by regulating the PI3K/Akt signaling pathway mediated by ABI2, western blotting assay was employed to confirm the presence of crucial proteins in this pathway. As shown in Fig. 8a-b, the findings demonstrated a significant inhibition of p-PI3K, p-Akt and p-mTOR expressions with both OE-ABI2 and CS pre-treatments. Furthermore, TUNEL (Fig. 8c-d) and wound-healing (Fig. 8e) assay results also indicated that CS and overexpressed ABI2 induced cell apoptosis and inhibited cell migration significantly. In addition, as shown in Fig. 8f, MDA-MB-231 cell apoptosis in CS- and OE-ABI2- groups was detected using an annexin V-FITC/PI assay, and the flow cytometry results indicated that the proportions of late apoptotic cells (Q2 area) in the CS group and the OE-ABI2 group were considerably greater than that in the Ctrl group. Hence, the aforementioned findings suggested that CS might induce TNBC cells apoptosis by inhibiting PI3K/Akt signaling pathway mediated by ABI2 overexpression.

## Discussion

Extensive evidence has indicated that the ubiquitin-protease system is intricately linked to the emergence and advancement of TNBC in the course of cancer progression, which can impede tumor growth by inducing autophagy, activating immune responses and eradicating the transformation of stem cells. Despite numerous studies in which the correlation between ubiquitination and TNBC is examined and dissected, there has been a lack of the ubiquitination-related database system utilized by researchers to screen and conduct further analyses on the significant regulatory function of ubiquitination substrate proteins in TNBC. Early on, using bioinformatics methods, fourteen differentially-expressed genes (RNF8, LEF1, PCNA, PLK2, CLCNS, TLR4, SGK1, TRAF6, ABI2, SIN3B, DTX3L, FIS1, STAP2, CASR) were selected and expressed specifically in the basal subtype of breast cancer (TNBC). A survival analysis of these genes showed that ABI2 were statistically different, which was conducive to the survival prognosis of TNBC. However, the function of ABI2 was unclear in TNBC, so we selected ABI2 as the key molecule for the follow-up studies.

To investigate the role of ABI2 protein in TNBC, we performed an IHC analysis on a tissue microarray consisting of 30 pairs of TNBC tumor tissues as well as their adjacent tissues in the current research. The IHC findings indicated that the ABI2 protein expression in TNBC tumors was markedly reduced compared to that in adjacent non-cancerous tissues. Western blotting results of in vitro experiments indicated a notable decrease in the expression of ABI2 protein in the TNBC cell line (MDA-MB-231) compared to normal breast cells (MCF-10 A). Furthermore, we verified the role of ABI2 protein in TNBC by conducting a range of in vitro phenotypic experiments. The outcome revealed that ABI2 had the ability to control cell cycles and suppress the migration, invasion as well as proliferation of MDA-MB-231 cells.

Furthermore, we performed RNA-seq analysis to investigate the particular molecular processes controlled by ABI2 in TNBC, and the results showed that 84 and 16 differential genes in OE-ABI2 group were involved in the PI3K/Akt and Hippo pathway, respectively. PI3K protein families, discovered 25 years ago in association with the altering ability of viral oncoproteins [19, 20], constitute a vast collection of lipid enzymes capable of phosphorylating the 3′-OH group of phosphatidylinositols (PtdIns) on outer cell membranes, resulting in the recruitment of macromolecule Ser/Thr-kinase and Akt to cell membranes, wherever it becomes activated. PI3K/Akt signaling cascade is aberrantly activated in several cancers and promotes the growth and survival of tumor cells [21–24]. So far, the two most common identified ways of activating PI3K/Akt in human cancers are initiated by receptor tyrosine kinases (RTKs) and genetic mutations of certain components of the signaling pathway [25]. Importantly, the activation of the PI3K pathway can adversely impact treatment strategies, thus favorable clinical outcomes could be yielded by blocking PI3K [26]. Hippo signaling pathway, first identified through investigations of drosophila melanogaster [27], is a conserved biological network that plays a crucial part in controlling cell growth. Hippo signaling pathway has been shown disrupted in various types of human cancers. Consistent with the function of growth inhibition, Hippo pathway generally acts as a tumor suppressor [28, 29].

The effects of ABI2 on PI3K/Akt and Hippo pathway were verified by qPCR, and the results showed that the expression changes of PI3K/Akt pathway related-genes were in accordance with the RNA-seq result. IP and LC-MS/MS results proved that ABI2 may regulate PI3K/Akt signaling pathway through interacting with RAC1 protein in TNBC cells. RAC1 is a GTPase and has multiple pathways to regulate PI3K/Akt signaling: (1) RAC1 protein combine with PI3K p110α isoform and thereby activate PI3K phosphorylation [30]; (2) RAC1 protein can bind to and inhibit PTEN expression, which is a negative regulatory factor of PI3K/Akt signaling network via the dephosphorylation of PI3-Kinase PIP(3) [31]. Co-IP experiment result finally confirmed the interaction between ABI2 and RAC1 proteins.

In order to elucidate the mechanism of ABI2 in TNBC, we used *Ubibrowser* database to predict the E3 ligases interacting with ABI2 protein. Co-IP and ubiquitination experiments confirmed that CBLC could promote the binding of ABI2 to Ub and increase the ubiquitination degradation of ABI2 protein. *BioGRID* database was used to verify the interactions between target proteins and target E3 enzymes. Further, we conducted molecular docking experiments to further determine the interaction pattern between ABI2 and CBLC proteins. The results showed that residues of CBLC and ABI2 interacted to form hydrophobic surfaces and hydrogen bonds that helped stabilize protein-protein complexes. Based on the above binding sites, we defined the structure of ABI2 as a receptor, with the binding site between ABI2 and CBLC proteins as a small molecule binding pocket site. Moreover, clinical trials of drugs have been completed by the FDA in general pharmacology, pharmacokinetics and toxicology, which lead FDA-based drug repositioning to be able to accelerate the drug research process. Therefore, we selected 2,800 FDA-approved drug molecules for our screening library, and Top 9 targeted small-molecule inhibitors were screened from the FDA drug compound library, and five compounds were evaluated through in vitro cell experiments for pharmacodynamics. The findings demonstrated that CS suppressed the proliferation of MDA-MB-231 cells in a dose-dependent fashion.

CS is a precursor drug hydrolyzed into polymyxin E, a polypeptide antibiotic produced by paenibacillus polymyxa, in human body and becomes effective [32]. The basic structure of polymyxin E is a heptapeptide ring connected by a tripeptide chain, the fatty acids in whose tail are connected to the end of the tripeptide by an amide bond [33]. Polymyxin E is mainly employed for the treatment of infections caused by gram-negative bacteria resistant to multiple drugs, whose antibacterial action involves cell membrane lysis, sac-sac interactions and the ability to induce oxygen stress, resulting in bacterial deaths [34, 35]. The most common side effects of polymyxin E are nephrotoxicity and neurotoxicity, involving possible cellular mechanisms including oxidative stress, apoptosis, cell cycle arrest and autophagy [36]. But so far, there have been no reports on the treatment of TNBC with polymyxin E mesylate sodium. Through in vitro experiments, we discovered that CS possessed the ability to hinder cell cycles, impede proliferation and restrict the migration of MDA-MB-231 cells through inhibiting PI3K/Akt signaling pathway. The findings of this research offer a conceptual foundation for the advancement of drug repositioning of polymyxin E, demonstrating commendable scholarly worth and practical importance.

In order to further verify whether CS works by regulating the PI3K/Akt signaling pathway mediated by ABI2, western blotting assay was employed to confirm the presence of crucial proteins in this pathway, and the results revealed that ABI2 and CS effectively suppressed the level of p-Akt, p-PI3K and p-mTOR. The PI3K/Akt/mTOR pathway is a crucial intracellular route that enhances cell proliferation, growth, tumorigenesis and motility [37, 38]. In short, the RTK-mediated activation of PI3K triggers Akt, and in response, overactivated Akt inhibits tuberous sclerosis complex (TSC), which serves as a GTPase-activating protein of Rheb, thereby activating mTOR, which faithfully picks up upstream activation signals to form two important mTOR complexes, mTOR Complex 1 (mTORC1) and mTOR Complex 2 (mTORC2), of which mTORC1 controls the process of protein production, metabolism and cellular expansion through the regulation of p70S6K1 and 4EBP1. In contrast, mTORC2 triggers the activation of Akt, which hinders the breakdown of cyclin D1/E. Inhibition of these activated signaling molecules can suppress tumor development [39]. In addition, certain phenotypic experiments conducted in vitro provided an additional confirmation that CS had the ability to suppress the PI3K/Akt signaling pathway through inhibiting CBLC mediated-ABI2 ubiquitylation.

**Fig. 9:**
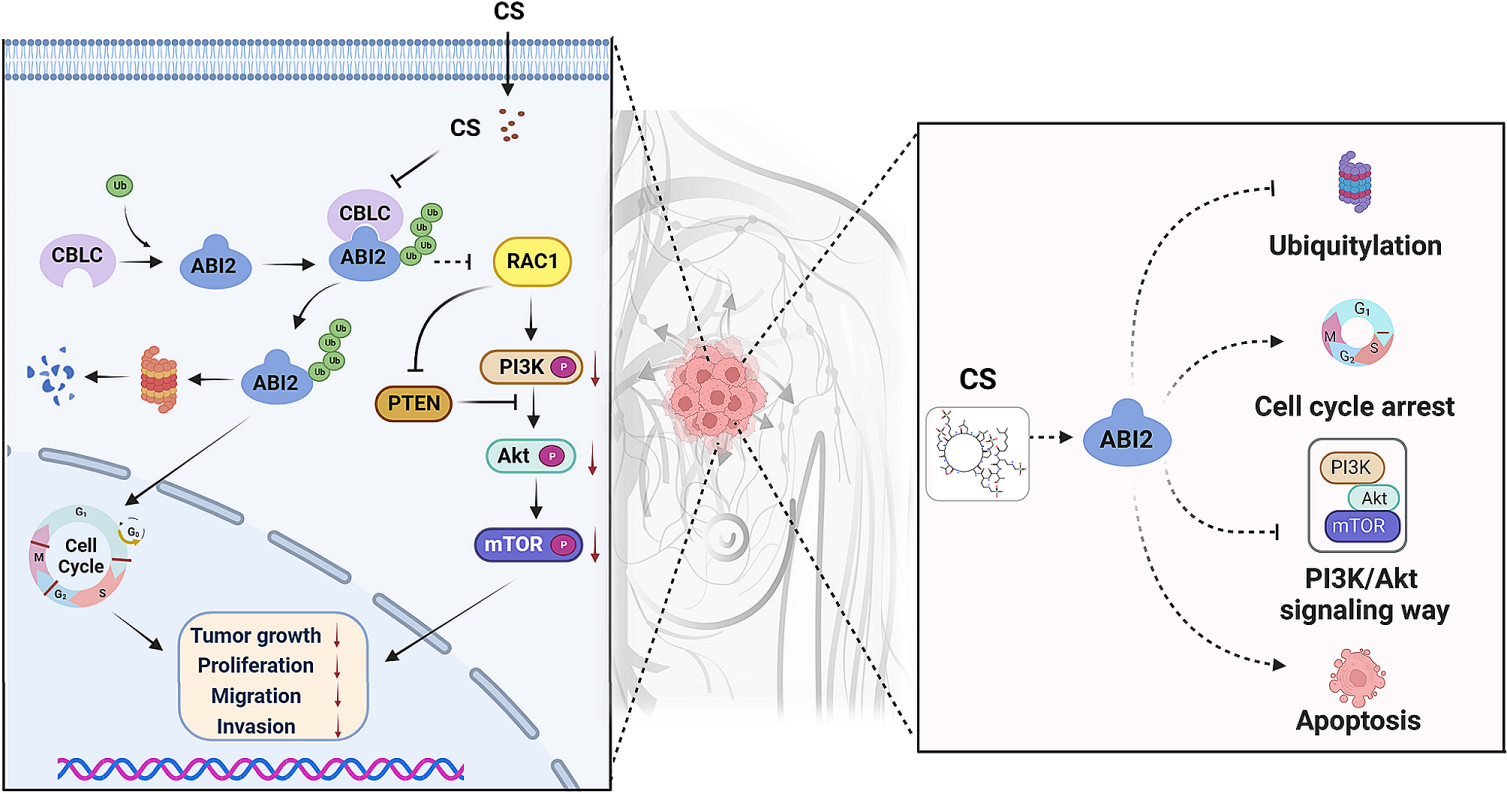
The molecular mechanism diagram of this study. ABI2, acting as a crucial factor of tumor suppression, hinders the initiation and progression of TNBC through inhibiting PI3K/Akt pathway via the interaction with RAC1 protein. Furthermore, the FDA-approved drug CS has shown significant potential in suppressing the proliferation of TNBC cells and inducing cell apoptosis by inhibiting the ubiquitination modification of ABI2 protein mediated by CBLC E3 ligase

To summarize, as shown in Fig. 9, ABI2, acting as a crucial factor of tumor suppression, hinders the initiation and progression of TNBC through inhibiting PI3K/Akt pathway via the interaction with RAC1 protein. Furthermore, the FDA-approved drug CS has shown significant potential in suppressing the proliferation of TNBC cells and inducing cell apoptosis by inhibiting the ubiquitination modification of ABI2 protein mediated by CBLC E3 ligase, making it a promising candidate for impeding the progression of TNBC. Of course, new favorable evidence for the development of CS drug repositioning for TNBC should be provided in vivo in the future.

### Electronic supplementary material

Below is the link to the electronic supplementary material.

**Supplementary Material 1**: **Figure S1** Heatmap analysis of the 279 ubiquitinated human substrate proteins expression in LumA, LumB and Her2 subtypes of breast cancer.

**Supplementary Material 2**: **Figure S2** (a) Molecular docking mode and amino acid interaction residues of ABI2 and CBLC proteins; (b) Molecular docking mode and amino acid interaction residues of ABI2 protein and CS.

## Data Availability

No datasets were generated or analysed during the current study.
