# Supplementary figures and images for: Inhibition of ABI2 ubiquitination-dependent degradation suppresses TNBC cell growth via down-regulating PI3K/Akt signaling pathway
Source: Cancer Cell Int. 2024 Jun 27;24:222. doi: 10.1186/s12935-024-03407-0 (PMC11212232; doi:10.1186/s12935-024-03407-0)

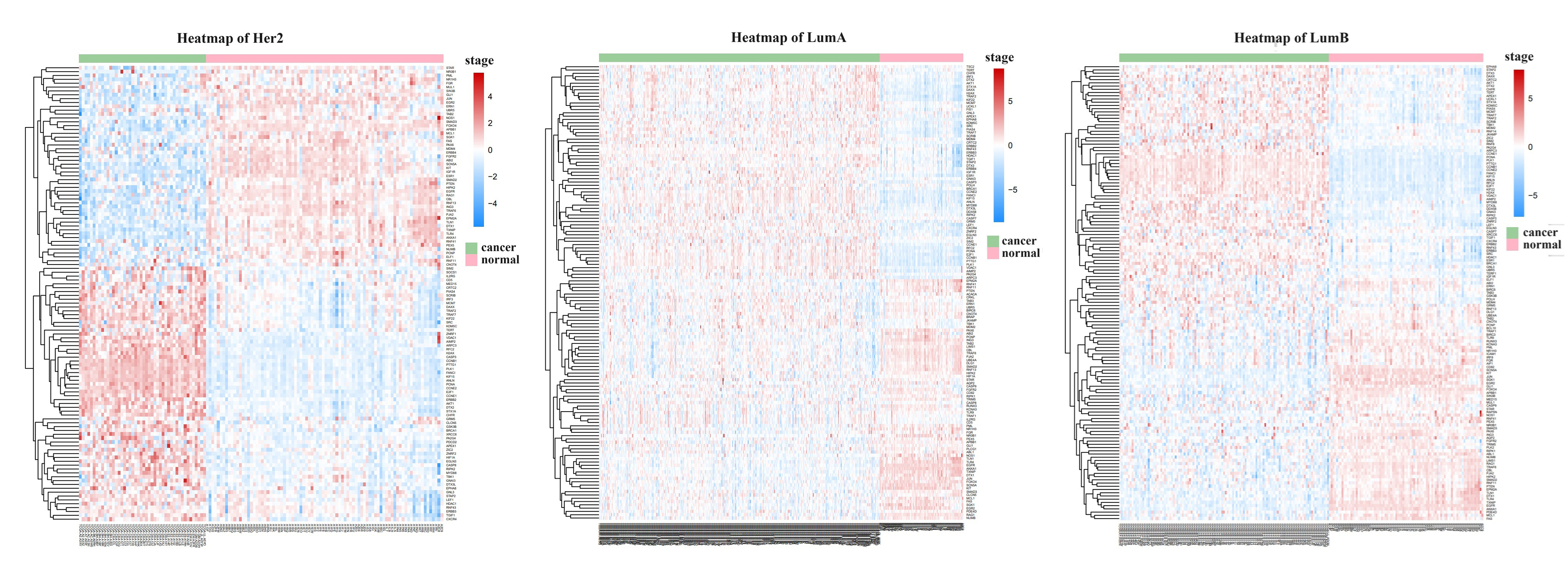

Supplement: Supplementary file 1 — Supplementary Material 1: Figure S1 Heatmap analysis of the 279 ubiquitinated human substrate proteins expression in LumA, LumB and Her2 subtypes of breast cancer. [file 12935_2024_3407_MOESM1_ESM.jpg]

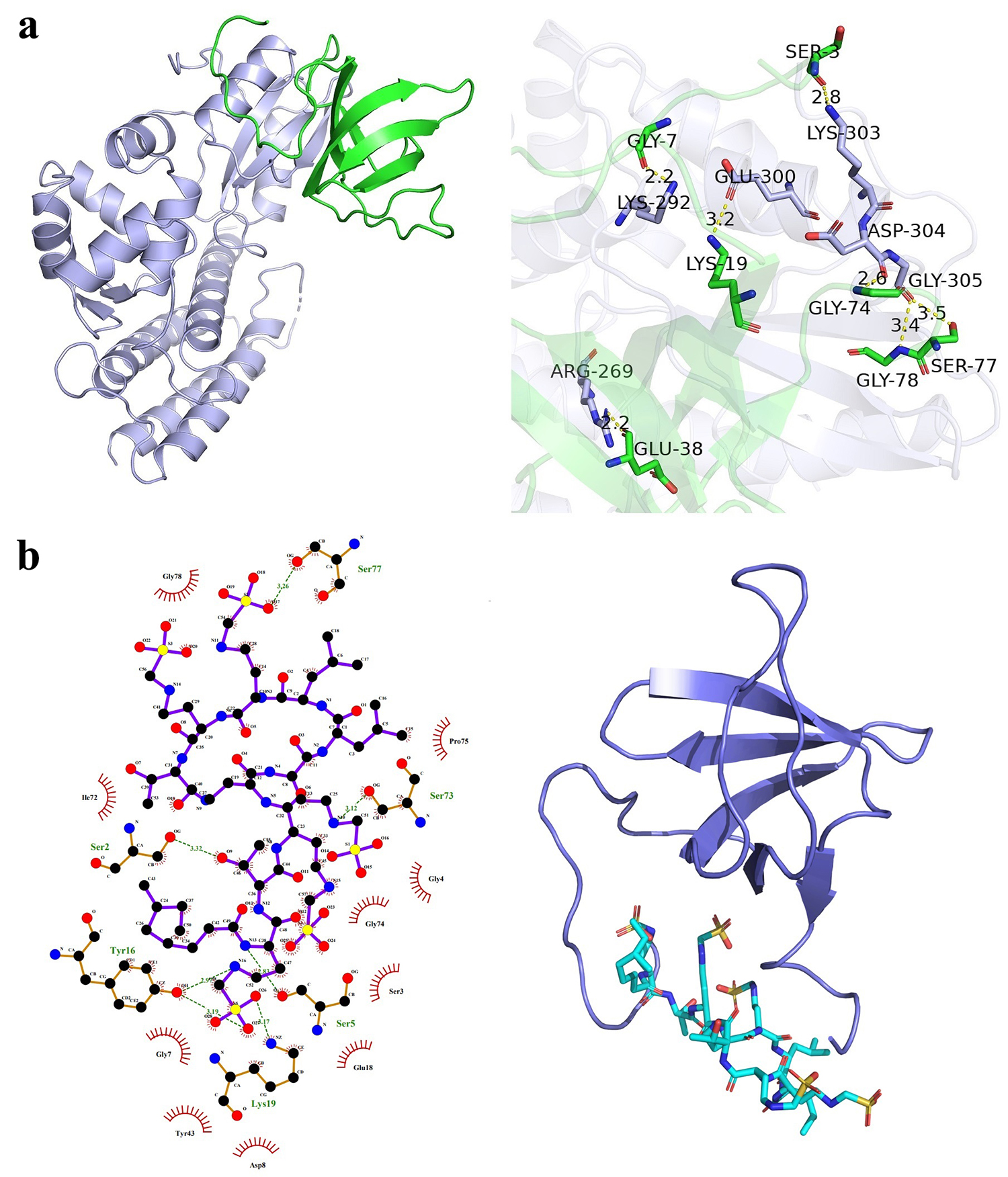

Supplement: Supplementary file 2 — Supplementary Material 2: Figure S2 (a) Molecular docking mode and amino acid interaction residues of ABI2 and CBLC proteins; (b) Molecular docking mode and amino acid interaction residues of ABI2 protein and CS. [file 12935_2024_3407_MOESM2_ESM.jpg]
